# Decidualised endometrial stromal cell‐derived extracellular vesicles induce bystander decidualisation and cAMP‐mediated attenuation of natural killer cell cytotoxicity

**DOI:** 10.1002/ctm2.70500

**Published:** 2025-10-11

**Authors:** Maryam Mousavi, Negar Vanaki, Kayhan Zarnani, Zahra Aghazadeh, Soheila Arefi, Jila Abedi‐Asl, Fazel Shokri, Seyed‐Alireza Razavi, Amir‐Hassan Zarnani

**Affiliations:** ^1^ Department of Immunology, School of Public Health Tehran University of Medical Sciences Tehran Iran; ^2^ Reproductive Biotechnology Research Center, Avicenna Research Institute Academic Center for Education Culture and Research Tehran Iran; ^3^ Reproductive Immunology Research Center, Avicenna Research Institute Academic Center for Education Culture and Research Tehran Iran; ^4^ Department of Tissue Engineering and Regenerative Medicine, Nanobiotechnology Research Center, Avicenna Research Institute, Academic Center for Education Culture and Research Tehran Iran

1

Dear Editor,

Decidualisation, the process of differentiation of endometrial stromal cells (EnSCs) into secretory decidual cells, is fundamental to blastocyst implantation and endometrial immune modulation. Defective decidualisation has been closely linked to implantation failure and miscarriage. Decidualisation triggers a metabolic and immunomodulatory shift in EnSCs, enabling them to regulate uterine natural killer (NK) cells and T cells.^1^

Understanding how decidual cell signalling influences neighbouring uterine cells is critical for elucidating key adaptations in early pregnancy, particularly maternal‒foetal crosstalk. A growing body of evidence highlights extracellular vesicles (EVs) as a novel axis of cell to cell communication, playing a pivotal role in tissue homeostasis and immune regulation.^2^ Here, we sought metabolic reprograming of EnSCs during decidualisation and unrevealed new aspects of endometrial EVs by showing that EVs from decidualised endometrial stromal cells (D‐EnSCs‐EVs) induce decidualisation in neighbouring cells and modulate NK cell function.

Here, EnSCs were isolated from luteal‐phase endometrial biopsies and characterised (Figure S1). Metabolome analysis of isolated cells demonstrated that decidualisation significantly alters the amino acid metabolome of EnSCs, effectively distinguishing undecidualised (uD) from decidualised (D) EnSCs by days 4 and 6 of decidualisation (Figure 1A,B). Cluster analysis revealed greater similarity in metabolomic profiles of late‐stage D‐EnSCs compared to earlier time points (Figure 1C). Metabolite analysis revealed methionine (Met) and phenylalanine (Phe) as key discriminators between uD‐EnSCs and D‐EnSCs (Figure 1D), and showed a coordinated shift in amino acid metabolism that may underpin the functional transformation of EnSCs during decidualisation (days 2–6; Figure 1E,F). Decidualised stromal cells are known to support decidual NK (dNK) cell generation from peripheral blood NK cells via secretion of transforming growth factor beta (TGF‐β), interleukin (IL)‐1β and IL‐15.^1^ Our finding that decidualisation induces methionine production provides a novel metabolic underpinning for this process, aligning with methionine's established epigenetic role in regulating IL‐5 transcription and promoting endometrial receptivity.^3^ Based on GLUT1 expression, it is thought that decidualisation relies on glucose metabolism.^4^ Our findings, however, demonstrated that the process was characterised by a reduction in glucose consumption and lactate production (Figure 1G), consistent with lower proliferation capacity of D‐EnSCs compared to uD‐EnSCs (Figure S2). Notably, high cyclic adenosine monophosphate (cAMP) concentration (.5 mM, as used in this study) suppress GLUT1 expression.^5^ Furthermore, decidualisation led to sustained pro‐inflammatory cytokines IL‐6 and IL‐8 secretion (Figure 1H), inversely correlated with the cells’ decidualisation capacity and prolactin secretion (Figure S2). These data align with the previous concept that decidualisation requires a transient inflammatory signal but is compromised by a prolonged inflammatory response.^6^

**FIGURE 1:**
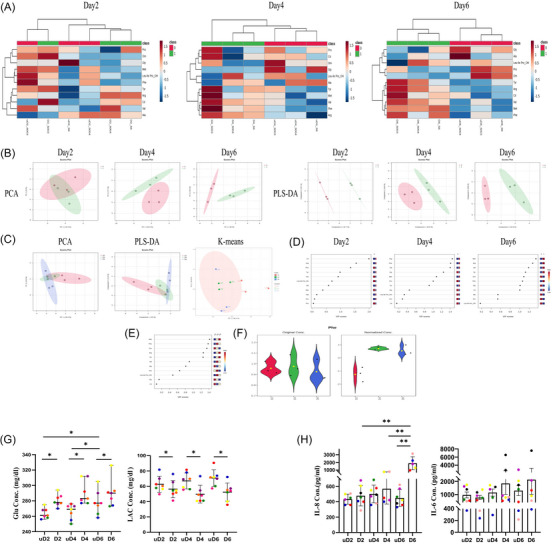
Metabolic profiling of decidualisation. Endometrial stromal cells (EnSCs) were in vitro decidualised using  .5 mM 8‐Br‐cAMP and 1 µM MPA for 2, 4 and 6 days. An amino acid metabolomics analysis was performed using liquid chromatography tandom mass spectrometry (LC‒MS/MS) (*n* = 3). (A) Heatmaps were used to visualise the results, illustrating differential metabolite expression between undecidualised (uD) (class 0 = red) and decidualised (D) (class 1 = green) cells and clustering the cells into two distinct groups on days 4 and 6 of decidualisation. The analysis revealed increased concentrations of amino acids such as Phe, Met, Val, Arg, Tyr and Cit, while levels of Orn, Gly and Pro decreased. (B) Principal component analysis (PCA) and partial least squares discriminant analysis (PLS‐DA) effectively distinguished the metabolomic profiles of uD (0 = red) and D (1 = green) cells. (C) Additionally, PCA, PLS‐DA and *K*‐means clustering were employed to differentiated the metabolomic profiles of D cells across days 2, 4 and 6 of decidualisation (D2 = red, D4 = green and D6 = blue). The findings revealed distinct clustering patterns between uD and D cells, as well as among D cells at different time points during decidualisation. (D) Variable importance in projection (VIP) score analysis was applied to the PLS‐DA results to identify the key amino acids influencing the metabolic transition between uD (class 0) and D cells (class 1). Based on the results, Met and Phe were crucial for discriminating between uD‐EnSCs and D‐EnSCs from days 2 to 6. (E) VIP score and (F) post hoc analyses were also used to refine clustering among D cells on days 2, 4 and 6 of decidualisation. Results indicated that Phe was the most determinate variable, showing an increasing trend during the progression of decidualisation. (G) Levels of glucose (Glu) and lactate (LAC) were measured in cell culture supernatants by colorimetric assay kits (*n* = 7). Based on the results, the glucose uptake and the lactate production were significantly decreased in D cells compared to their uD cells counterparts on days 2, 4 and 6 of decidualisation. (H) Levels of interleukin (IL)‐8 and IL‐6 were determined in cell culture supernatants by enzyme‐linked immunosorbent assay (ELISA) (*n* = 7). Results showed a steady‐state increase in the secretion of IL‐6 and IL‐8 by D cells on days 2 to 6. No significant difference was observed in IL‐6 secretion between uD and D cells; however, day 6 D cells secreted more IL‐8 compared to that of uD cells. *p*‐Values: less than ^*^.05, ^**^.01, ^***^.001 and ^****^.0001.

To explore the potential role of EnSC‐derived EVs on induction of decidualisation, EVs were isolated (Figure S3 and Table S1) from D‐EnSCs and uD‐EnSCs and characterised (Figure 2A,B). Decidualisation significantly increased EV secretion (Figure 2C). Subsequent uptake kinetics assays showed a cell‐type‐specific pattern. EnSCs internalised EVs rapidly (within 4 h; Figure 2D,E) in contrast to NK cells, which displayed a slower uptake profile, requiring 24 h for significant incorporation (Figure 2F). This temporal difference may reflect variations in size‐dependent endocytic activity between the two cell types.

**FIGURE 2:**
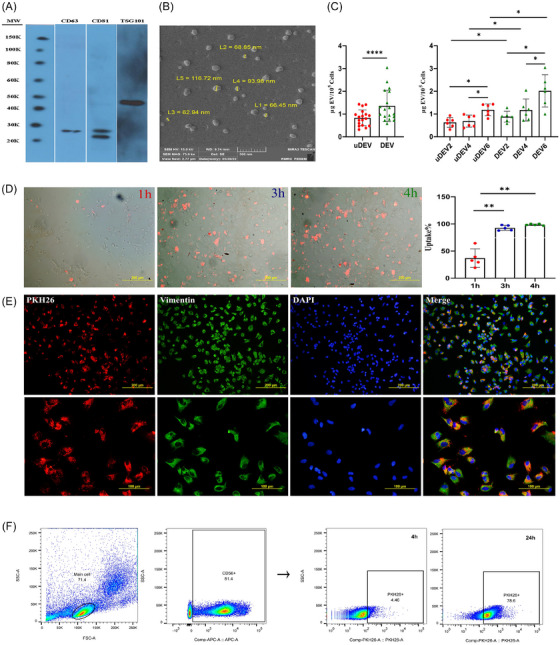
Characterisation and internalisation of extracellular vesicles (EVs) derived from endometrial stromal cells (EnSCs). EVs were isolated from EnSCs and characterised. (A) Western blot analysis confirmed the presence of EV markers (CD63, TSG101 and CD81), and (B) field emission scanning electron microscopy (FE‐SEM) provided high‐resolution images showing the spheroid ultrastructure and size of EVs, ranging from 62 to 116 nm. Scale bar: 500 nm. (C) EnSCs were subjected to in vitro decidualisation (IVD), and EVs were isolated from both undecidualised (uD) and decidualised (D) cells on days 2, 4 and 6 of decidualisation (uDEV2, 4, 6 and DEV2, 4, 6) to evaluate their concentration dynamics during IVD. Our results showed that regardless of the differentiation day, D cells produced higher amounts of EVs compared to uD cells (*n* = 6). EVs were labelled with PKH‐26 dye and the kinetic of EVs uptake by EnSCs and natural killer cells (NKs) was assessed. (D) Incubation of PKH26‐labelled EVs with EnSCs for 1‒4 h resulted in EV uptake by EnSCs (*n* = 5). Scale bars: 200 µm. (E) A three‐colour immunostaining using PKH‐26 (red), vimentin (green) and DAPI (blue) was performed on EnSCs co‐cultured with EVs. After a 4‐h incubation period, nearly all EnSCs captured PKH26‐labelled EVs. Scale bars: 200 µm (upper panel) and 100 µm (lower panel). (F) EVs uptake by NKs was also evaluated after 4 and 24 h using flow cytometry. The results showed that a minority of NK cells captured EVs after 4 h, which was increased to about 78% after a period of 24‐h incubation. *p*‐Values: less than ^*^.05, ^**^.01, ^***^.001 and ^****^.0001.

To extend prior findings^4^ that EVs induce decidualisation genes in EnSCs, we next characterised the kinetics of this process at the protein level and under various culture conditions. These effects were evaluated both with EVs alone and in combination with various decidualisation inducers, across multiple EV collection time points. At all tested concentrations, EVs derived from decidualised EnSCs (DEVs) consistently demonstrated a robust capacity to induce decidualisation compared to EVs from undecidualised EnSCs (uDEVs), under various culture conditions on days 3 and 6 (Figure 3A,C). A marked effect was observed with EVs isolated on day 4 of decidualisation (DEV4) (Figure 3D‒F). These results underscore the time‐sensitive nature of EV composition during decidualisation and point to DEV4 as an optimal pro‐decidualisation signal. Interestingly, higher concentrations of differentiation stimuli paradoxically reduced decidualisation efficiency (Figure S2). Notably, prolactin secretion by DEVs was inhibited on days 3 and 6 under optimal concentration of cAMP and medroxyprogesterone acetate (MPA), indicating a threshold beyond which excessive signalling may disrupt cellular function (Figure 3B). Accordingly, similar to DEV4 derived from primary EnSCs, DEV4 isolated from an endometrial stromal cell line (ENSC) also induced decidualisation in primary EnSC cultures, albeit with lower potency (Figure 3G). Unlike uDEV from some donors, which modestly elevated prolactin secretion, uDEV4 derived from human foreskin fibroblasts (FSK‐EV4) failed to induce decidualisation (Figure 3H), underscoring that EV functionality depends on cellular origin.

**FIGURE 3:**
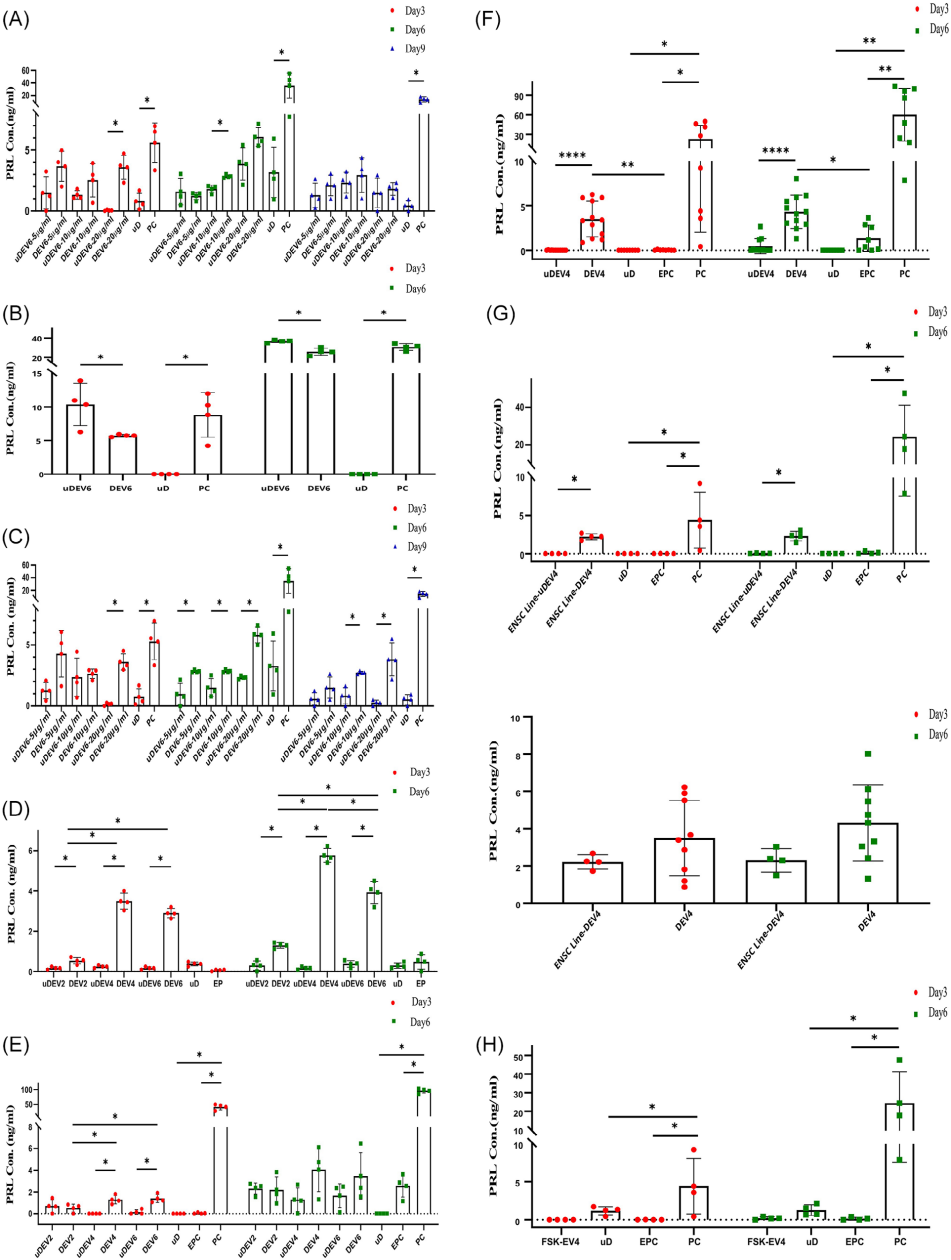
Induction of endometrial stromal cells (EnSCs) decidualisation by EnSCs‐EVs at different culture conditions. EnSCs were in vitro decidualised using  .5 mM 8‐Br‐cAMP and 1 µM MPA (D) or remained undifferentiated (uD) for 2, 4 and 6 days. Supernatants from both groups were collected, and extracellular vesicles (EVs) were extracted using the EXOCIB C kit. EnSCs were co‐cultured with uD or D cell‐derived EVs (uDEV and DEV) from days 2–6 of decidualisation to assess their pro‐decidualisation effect on undecidualised EnSCs via prolactin measurement. This effect was tested at different settings, where EnSCs were cultured either in basal medium or primed with various decidualisation stimulants. (A) The effect of EVs derived from in vitro decidualised EnSCs for 6 days (uDEV6 and DEV6) on EnSCs’ decidualisation was assessed on days 3, 6 and 9 at different concentrations of EVs in basal medium. DEVs induced higher prolactin secretion on days 3 and 6 compared to uDEVs. (B) The impact of uDEV6 and DEV6 on decidualisation of EnSCs cultured under Progesterone + cAMP (PC) condition was measured on days 3 and 6. Treatment with DEVs, in the presence of optimal concentrations of cAMP and MPA, inhibited prolactin secretion at both time points. To evaluate the effect of EVs in the context of decidualisation stimuli and the potential for synergistic interaction, their impact was assessed in the presence of progressively increasing strengths of decidualisation‐inducing stimuli, ranging from weak to strong stimuli. (C) The effect of uDEV6 and DEV6 was assessed on days 3, 6 and 9 of EnSCs culture in the presence of 1 µM MPA. DEVs demonstrated a consistently stronger pro‐decidualisation effect than uDEVs across all EV concentrations and time points. (D) Decidualisation is a process that shifts from a pro‐inflammatory to an anti‐inflammatory state. Based on this concept, it was assessed whether EVs isolated during different days of decidualisation exert a differential effect on the induction of decidualisation in EnSCs. The effects of DEV2‐6 on EnSCs decidualisation were assessed under Estradiol + Progesterone (EP) stimulation. DEV4 induced higher prolactin secretion compared to DEV2 and DEV6, and significantly higher levels compared to when EnSCs were treated with E2 + MPA. (E) The pro‐decidualisation effect of DEV2‐6 under Estradiol + Progesterone + cAMP (EPC) stimulation again showed the superior pro‐decidualisation effect of DEV4 compared to the DEV2 and DEV6. (F) Based on the promising results (E), the pro‐decidualisation effect of DEV4 were further tested on undecidualised EnSCs derived from 12 donors and it was confirmed that DEV4 not only significantly induce decidualisation in EnSCs both on days 3 and 6 compared to uDEV4 but also their pro‐decidualisation effect is superior to the standard protocol of decidualisation medium containing E2 + MPA + cAMP. (G) To gain insight into the pro‐decidualisation effect of EVs derived from an endometrial stromal cell line (ENSC), DEV4 from ENSC cells were tested in the same setting. The impact of DEV4 derived from primary EnSCs (DEV4) and ENSC Line‐derived DEV4 (ENSC Line‐DEV4) on decidualisation of EnSCs under EPC stimulation was compared. ENSC Line‐DEV4 were found to be able to induce decidualisation, albeit with a lower potency. (H) It was observed that uDEV from some donors could induce low levels of prolactin, although their potency to induce decidualisation was significantly lower than their corresponding DEVs. In this regard, EVs from foreskin (FSK‐EV4) cultured with EnSCs and their effect on prolactin secretion in undecidualised EnSCs under EPC simulation was assessed. Neither on day 3 nor on day 6, FSK‐EV4 had a pro‐decidualisation effect. PC: 1 µM MPA +  .5 mM cAMP, EP: 10 nM E2 + 1 µM MPA, EPC: 10 nM E2 + 1 µM MPA +  .05 mM cAMP. *p*‐Values: less than ^*^.05, ^**^.01, ^***^.001 and ^****^.0001.

Although these experiments were conducted in vitro, the presence of EVs in human uterine fluid and their potential role in modulating the maternal‒foetal interface has been reported earlier. It is noteworthy that uterine fluid extracellular vesicles (UF‐EVs) mirror the dynamic mRNA and miRNA changes of the endometrial tissue across the menstrual cycle.^7^ Therefore, the functional effects we observed, along with previous findings that EnSCs‐EVs enhance vascular network formation and stimulate trophoblast differentiation,^4^ likely reflect the natural role of EVs at the maternal‒foetal interface. Consequently, analysing endometrial EVs shows significant promise as a basis for non‐invasively assessing endometrial decidualisation potential.

Decidualisation and modulation of endometrial immune cells are closely linked processes. Within this context, modulating the function of NK cells, the most prevalent immune population in the early pregnant endometrium, is critically important, as they are essential regulators for maintaining pregnancy.^8^ Notably, menstrual stromal cells (MenSCs), commonly used as EnSC surrogates, can shift NK cells towards a dNK‐like phenotype.^9^ Examining the effect of EVs on NK cell function, revealed that EVs derived from D‐EnSCs did not significantly influence NK cell proliferation (Figure 4A), they notably, however, attenuated NK cell cytotoxicity against both EnSCs and K562 target cells (Figure 4B). Moreover, although treatment with these EVs did not significantly affect the expression of NK cell phenotypic and functional markers (Figure 4C), EVs from D‐EnSCs increased the frequency of CD56^bright^ NK cells (Figure 4D), indicating a shift towards a ‘pregnancy‐friendly’ phenotype and the role of D‐EnSCs‐EV in shaping uterine immune responses. Interestingly, decidualisation mediators (cAMP + MPA) exerted the same effects on the NK cell cytotoxicity and the frequency of CD56^bright^ NK cells (Figure 4B,D) suggesting that the impact of D‐EnSCs‐EV on NK cell cytotoxicity is in part mediated by decidualisation mediators (cAMP) that are packaged into the EVs during the decidualisation process. Interestingly, cAMP has been shown to modulate the function of NK cells by promoting the emergence of CD56^bright^ NK subsets, as we showed here, and this effect is mediated, at least in part, through the activation of the transcription factor FOXO1, a key regulator of NK cell differentiation within the decidual microenvironment.^10^

**FIGURE 4:**
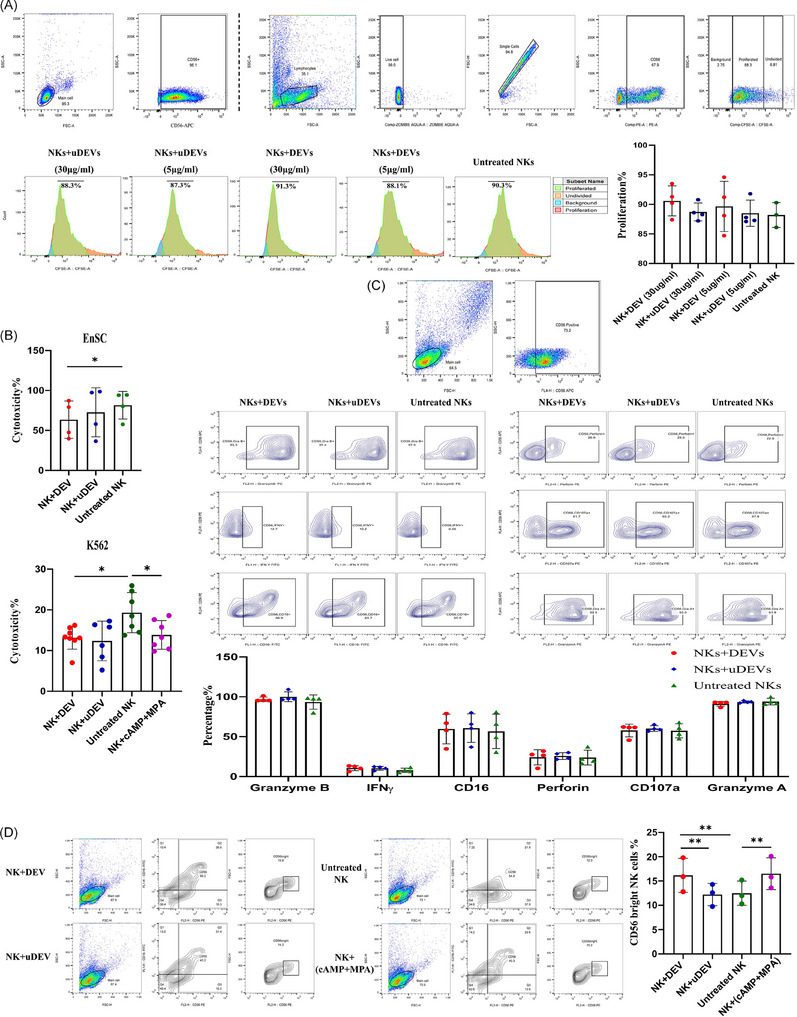
Effect of endometrial stromal cells (EnSC)‐derived extracellular vesicles (EVs) on natural killer (NK) cell proliferation, cytotoxicity and expression of phenotypic and functional markers. NK cells were isolated from peripheral blood mononuclear cells (PBMCs) and the effect of EnSC‐derived EVs on their proliferation (A), cytotoxicity on EnSCs and K562 cells (B) and phenotypic and functional markers (C and D), were measured by carboxyfluorescein succinimidyl ester (CFSE) flow cytometry, calcein‐AM (C‐AM) fluorimetry and flow cytometry using specific markers, respectively. (A) NK cells were labelled with CFSC and treated with EVs derived from IFN‐γ pre‐treated undecidualised (uD) and decidualised (D) EnSCs on day 4 of decidualisation (uDEV4 and DEV4), or remained untreated (untreated NK). NKs proliferation was assessed after 5 days of incubation in the presence of IL‐15 (10 ng/mL). Upper left panel shows flow cytometry results of NK cell purity after MACS separation. Upper right panel shows gating strategy in NK cell proliferation assay. uDEVs or DEVs did not exert a significant effect on the proliferation of NK cells. (B‒D) In subsequent assays, NK cells were treated with EVs derived from IFN‐γ pre‐treated undecidualised (uD) and decidualised (D) EnSCs on day 4 of decidualisation (uDEV4 and DEV4), treated with decidualisation mediators (.5 mM cAMP and 1 µM MPA), or remained untreated for 4 days in the presence of IL‐15. (B) For cytotoxicity assessment, EV‐treated, decidualisation mediator‐treated, or untreated NK cells were co‐cultured either with C‐AM−labelled K562 cells for 4 h or with EnSCs for 72 h. After the 72‐h co‐culture, NK cells were removed, and adherent EnSCs were subsequently labelled with C‐AM. Fluorescence intensity in culture supernatants or cell lysates was measured using a fluorimeter. Based on results, NK cells treated with DEV (NK + DEV), compared with untreated NK cells, showed a reduced capacity to exert cytotoxic effects on both EnSCs and K562 cells. Similarly, decidualisation mediator‐treated NK cells exhibited decreased cytotoxicity against K562 cells. Results are presented as mean ± SD. (C) On day 4, EV‐treated NK cells were co‐cultured with K562 cells for 4‐h incubation followed by marker analysis. Accordingly, uDEV4 or DEV4 did not exert a significant effect on granzyme A/B, perforin, CD107, IFN‐γ and CD16 expression compared to untreated NK cells (*n* = 4). (D) However, treating NK cells with DEVs significantly increased the percentage of CD56^bright^ cells compared to NK cells treated with uDEVs and untreated NK cells. Notably, decidualisation mediators similarly increased the proportion of CD56^bright^ NK cells. Results are expressed as mean ± SD. *p*‐Values: less than ^*^.05, ^**^.01, ^***^.001 and ^****^.0001.

These findings are also consistent with our observation that decidualisation of EnSCs upregulated HLA‐G expression, known to prevent NK cell cytotoxicity, following interferon gamma (IFN‐γ) pre‐treatment (Figure S4), and with the previous reports demonstrating that pro‐inflammatory stimulation of stromal cells with IFN‐γ and tumor necrosis factor alpha (TNF‐α) enhances the anti‐inflammatory and immunomodulatory properties of EVs.^11^

To confirm these in vitro findings, in vivo studies are necessary. This would involve evaluating decidualisation markers in paired samples of UF‐EVs and endometrial tissues, a crucial step for developing EV‐based assessments of decidualisation capacity in women with infertility or miscarriage. Recent studies have also highlighted the diagnostic and therapeutic potential of EVs in endometrial pathologies such as endometriosis,^12^ further supporting the relevance of EV‐associated biomarkers in identifying endometrial dysfunction and guiding future interventions.

In conclusion, this research offers key insights into molecular interactions at the maternal‒foetal interface, emphasising the essential role of EVs in endometrial decidualisation and immune regulation, and proposes EVs as potential diagnostic agents for reproductive failures associated with impaired decidualisation.

## AUTHOR CONTRIBUTIONS

Maryam Mousavi performed all experiments and wrote the first draft of the manuscript. Negar Vanaki contributed in performing the experiments and R data analysis. Kayhan Zarnani and Zahra Aghazadeh contributed in performing the experiments. Soheila Arefi and Jila Abedi‐Aal acted as gynaecologist advisors and provided the endometrial biopsies. Fazel Shokri and Seyed‐Aliraza Razavi critically read and edited the final version of the manuscript. Amir‐Hassan Zarnani extensively contributed in conceptualisation, project administration, supervision, data validation, writing and critically editing the manuscript.

## CONFLICT OF INTEREST STATEMENT

The authors declare they have no conflicts of interest.

## FUNDING INFORMATION

The financial support was received for this research by grants from the Iranian Council for the Development of Regenerative Medicine and Stem Cell Technologies (https://stemcell.isti.ir/) (grant no. 11/104553) and Tehran University of Medical Sciences (https://en.tums.ac.ir/en) grant (no. 1401‐1‐99‐57053).

## ETHICS STATEMENT

All procedures carried out within the scope of this study received ethical approval from the Tehran University of Medical Sciences (TUMS) ethics committee under the reference code IR.TUMS.SPH.REC.1401.015. Written informed consent was obtained from all participants prior to inclusion in the study.

## Supporting information



Supporting Information

Supporting Information

Supporting Information

Supporting Information

Supporting Information

Supporting Information

Supporting Information

## Data Availability

The data that support the findings of this study are available from the corresponding author upon reasonable request.
