# Supplementary figures and images for: Decidualised endometrial stromal cell‐derived extracellular vesicles induce bystander decidualisation and cAMP‐mediated attenuation of natural killer cell cytotoxicity
Source: Clin Transl Med. 2025 Oct 11;15(10):e70500. doi: 10.1002/ctm2.70500 (PMC12514555; doi:10.1002/ctm2.70500)

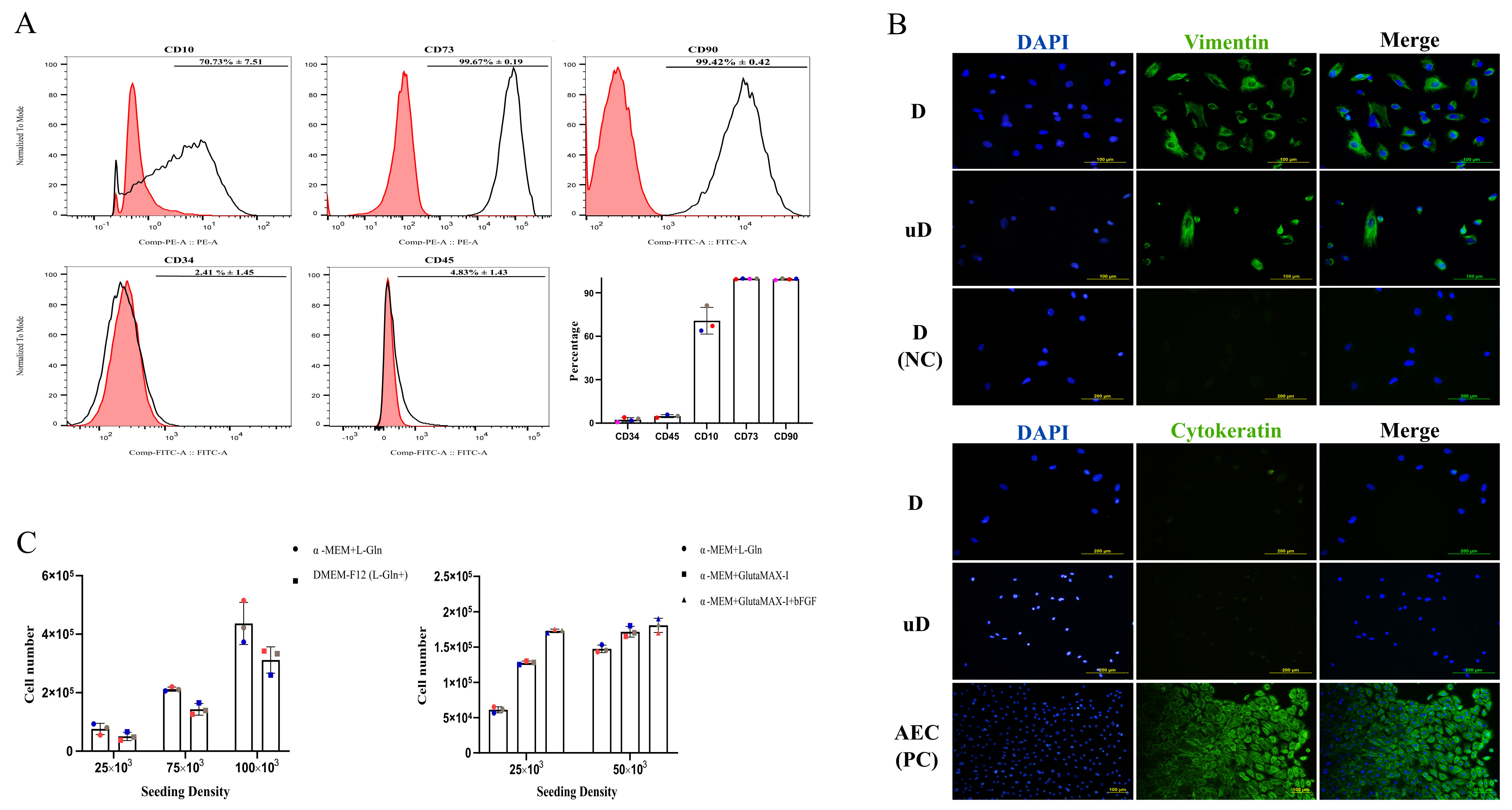

Supplement: Supplementary file 1 — Supporting Information [file CTM2-15-e70500-s001.jpg]

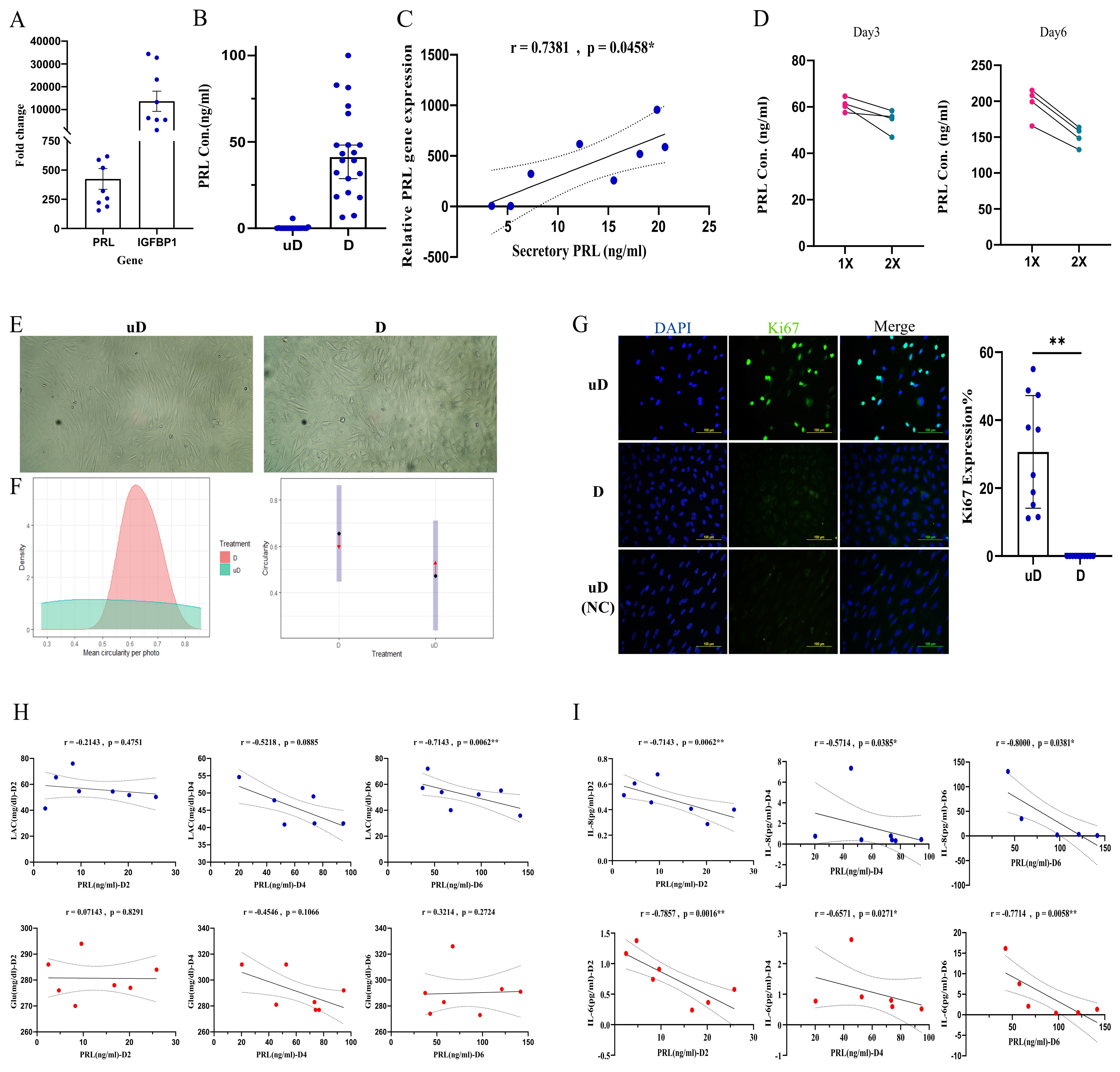

Supplement: Supplementary file 2 — Supporting Information [file CTM2-15-e70500-s006.jpg]

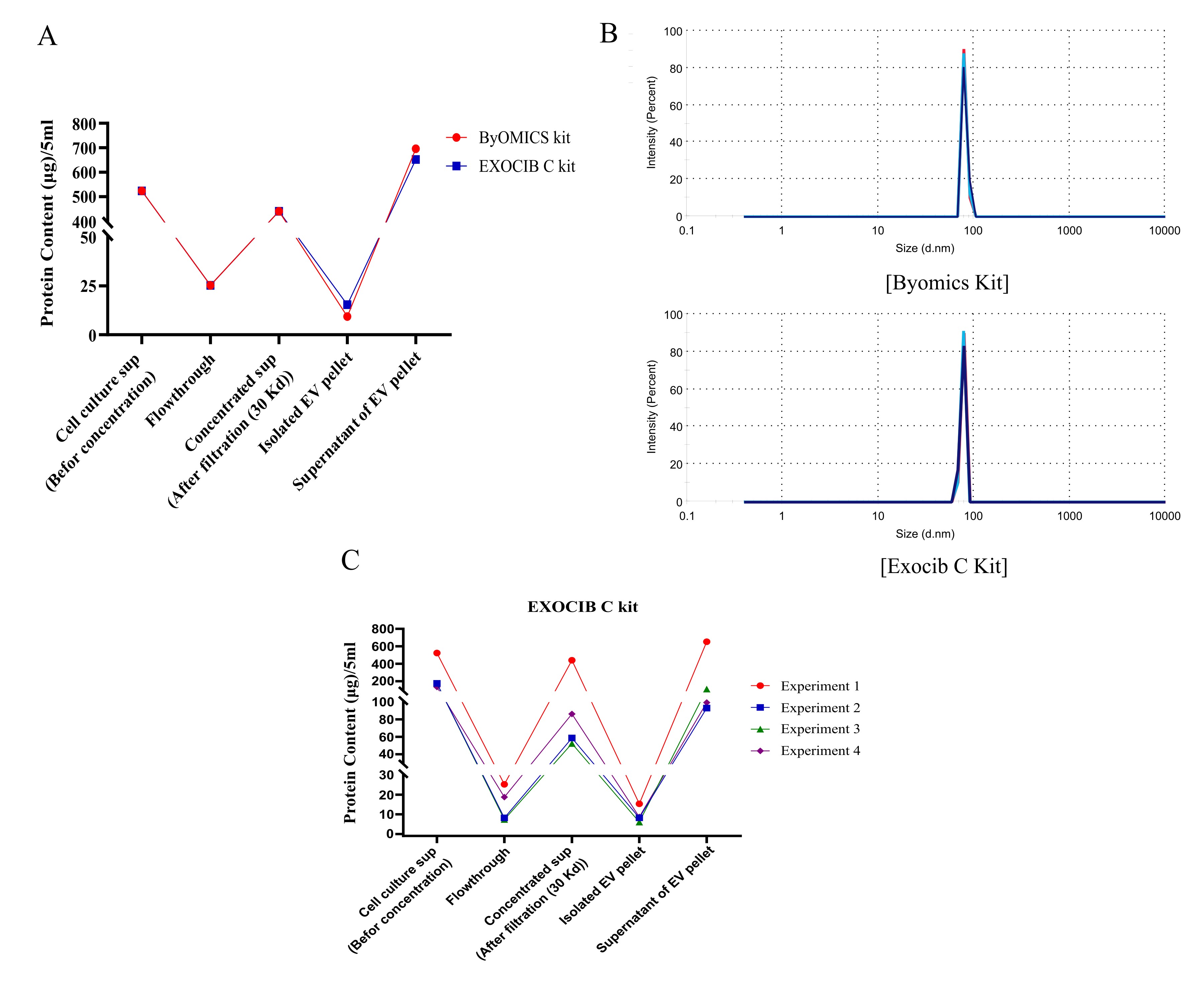

Supplement: Supplementary file 3 — Supporting Information [file CTM2-15-e70500-s007.jpg]

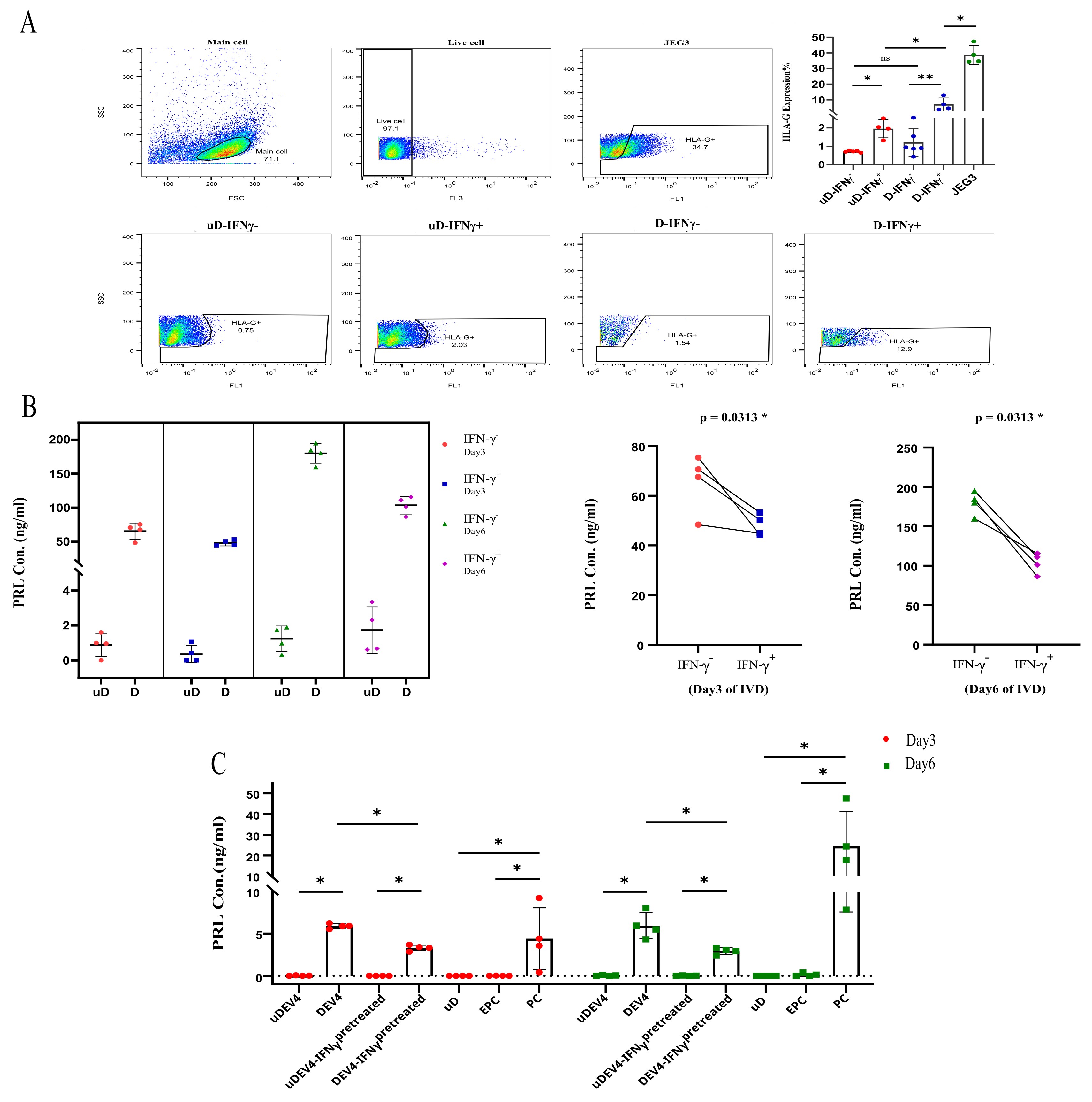

Supplement: Supplementary file 4 — Supporting Information [file CTM2-15-e70500-s005.jpg]
