# Supplementary material for: Decidualised endometrial stromal cell‐derived extracellular vesicles induce bystander decidualisation and cAMP‐mediated attenuation of natural killer cell cytotoxicity
Source: Clin Transl Med. 2025 Oct 11;15(10):e70500. doi: 10.1002/ctm2.70500 (PMC12514555; doi:10.1002/ctm2.70500)
Supplement: Supplementary file 5 — Supporting Information [file CTM2-15-e70500-s002.docx]

**Supplementary Information**

**Decidualized Endometrial Stromal Cell-Derived Extracellular Vesicles Induce Bystander Decidualization and cAMP-mediated Attenuation of Natural Killer Cell Cytotoxicity**

Maryam Mousavi ^1^, Negar Vanaki ^1^, Kayhan Zarnani ^1^, Zahra Aghazadeh ^1^, Soheila Arefi ^2^, Jila Abedi-Asl ^2^, Fazel Shokri ^1^, Seyed-Alireza Razavi ^1^, Amir-Hassan Zarnani ^1,3,4^

*^1^ Department of Immunology, School of Public Health, Tehran University of Medical Sciences, Tehran, Iran.*

*^2^ Biotechnology Research Center, Avicenna Research Institute, Academic Center for Education, Culture and Research (ACECR), Tehran, Iran.*

*^3^ Reproductive Immunology Research Center, Avicenna Research Institute, Academic Center for Education, Culture and Research (ACECR), Tehran, Iran.*

*^4^ Department of Tissue Engineering and Regenerative Medicine, Nanobiotecnology Research Center, Avicenna Research Institute, Academic Center for Education, Culture and Research (ACECR), Tehran, Iran.*

***Correspondence**: Amir-Hassan Zarnani

Department of Immunology, School of Public Health, Tehran University of Medical Sciences, Tehran, Iran, and Department of Tissue Engineering and Regenerative Medicine, Nanobiotecnology Research Center, Avicenna Research Institute, Academic Center for Education, Culture and Research (ACECR), Tehran, Iran.

**Tel.:** +982144933132

**Email:** [zarnaia@sina.tums.ac.ir](mailto:zarnaia@sina.tums.ac.ir), [zarnania@gmail.com](mailto:zarnania@gmail.com)

**Supplementary Materials and Methods**

**Acquisition of cells and tissues**

This research involved women who were admitted to an IVF facility for infertility treatment. These participants underwent hysteroscopy conducted by an obstetrics and gynecology (OB-GYN) expert for diagnostic purposes, and endometrial biopsy samples were collected. Following the adherence to specific inclusion criteria (women aged 20 to 40 in the luteal phase of their menstrual cycle) and exclusion criteria (vaginal discharge, presence of bacterial or viral infections, positive laboratory results for HBV, HCV, or HIV, history of autoimmune diseases, endometriosis, recent chemotherapy within the last three years, use of corticosteroids or hormonal treatments within the last three months), a total of twenty patients referred to an IVF clinic were selected to participate in this research study. The samples were transported to the laboratory using a transport medium containing **Dulbecco's modified eagle medium/nutrient mixture F12 (**DMEM-F12) supplemented with antibiotics (penicillin G at 100 IU/ml and streptomycin at 0.1 mg/ml) along with 0.25 μg/ml of amphotericin B) in a cold chain. Based on the decidualization potential, the experiments were conducted on sources with prolactin (PRL) secretion equal to or above the median (n=12).

The endometrial stromal cell line (ENSC) was sourced from the Iranian Biological Resource Center (IBRC). The cells were cultured in DMEM-F12 medium supplemented with 10% fetal bovine serum (FBS) and antibiotics at 37°C in a humidified incubator with 5% CO2.

Primary cultures of foreskin-derived mesenchymal stromal cells were prepared from the prepuce of male children undergoing circumcision. Human amniotic-membrane-derived epithelial cells were obtained from the cell bank of the pathobiology department, the School of Public Health, Tehran University of Medical Sciences (TUMS). The human choriocarcinoma cell line, JEG3, was a gift from Dr. Nassim Ghaffari Tabrizi-Wizsy, Graz Medical University. All procedures carried out within the scope of this study received ethical approval from the Tehran University of Medical Sciences (TUMS) ethics committee under the reference code IR.TUMS.SPH.REC.1401.015. Written informed consent was obtained from all participants prior to inclusion in the study.

**Human endometrial stromal cell isolation and characterization**

The process of isolating human endometrial stromal cells involved mincing endometrial samples into tiny fragments using a scalpel. These fragments were then digested in a digestion medium, DMEM-F12 containing 0.5 mg/ml collagenase and 0.05 mg/ml DNase, for 1 hour at 37°C. During this time, the mixture was agitated every 20 minutes. Enzyme activity was blocked by adding an equivalent amount of DMEM-F12 with 10% FBS, and cell count and viability were determined.  Cells were cultured, and the adherent cells were collected and passaged. Expression of cell surface markers was assessed by direct flow cytometry using PE-labeled mouse anti-human CD10 (BD Biosciences, USA, cat no: 555375), CD73 (BioLegend, USA, cat no: 344004), FITC-labeled mouse anti-human CD90, CD34 and CD45 (BioLegend, cat no: 328108, 343604, and 304006, respectively) on the EnSCs detached by Citrate buffer. The stromal nature of the isolated cells was further confirmed by immunofluorescent staining of isolated cells. The immunofluorescent labeling process involved fixing cultured cells in ice-cold acetone and staining with FITC-conjugated mouse anti-human cytokeratin (BD Biosciences, cat no: 347653). A mouse anti-human vimentin antibody (Santa Cruz, USA, cat no: SC-6260) was applied for vimentin staining, followed by FITC-conjugated sheep anti-mouse immunoglobulin (Sina Biotech, Iran). Nuclear staining was accomplished using DAPI (Sigma, USA).

**Optimization of EnSCs culture condition**

**EnSCs were isolated and cultured in a** 12-well culture plate with varying seeding densities (25, 75, and 100 × 10^3^) in either DMEM-F12 containing L-glutamine or α-MEM medium and L-glutamine (2 mM), both supplemented with 10% FBS and antibiotics (**Penicillin G 100IU/ml, Streptomycin 0.1mg/ml). The cells were then incubated at 37°C in a humidified 5% CO2 incubator. After 5 days, cell count and viability were determined. Based on the results, α-MEM was selected for further experiments. EnSCs were then cultured at** two different seeding densities (25 and 50 × 10^3^) **in α-MEM-FBS 10% containing either** 2 mM **L-glutamine, GlutaMAX (Gibco, USA), or GlutaMAX and** basic fibroblast growth factor **(b-FGF) (10 ng/ml) (Sigma). This incubation was continued for 5 days, after which the cells were washed with phosphate-buffered saline (PBS), detached using 0.25% trypsin-EDTA, and counted.** Based on the results, cell culture condition that supported the highest rate of proliferation of ESCs within the shortest timeframe was selected. No cell source with a passage number greater than five was used in the experiments. Multiple stocks were generated and frozen from each cell source.

***In vitro* Decidualization (IVD) and characterization**

For *in vitro* decidualization (IVD), isolated EnSCs or human endometrial cell line (ENSC) were propagated in T-75 culture flasks in α-MEM medium supplemented with 10% FBS, GlutaMax, and b-FGF (10 ng/ml). After reaching 70% confluency, the cells were washed with a warm medium, and a new medium containing DMEM-F12 supplemented with 10% charcoal-stripped (CS)-FBS was added. Once the cells achieved approximately 80% confluency, the media was switched to a resting medium (DMEM-F12 without phenol red, 2% CS-FBS), and the cells were incubated overnight. Subsequently, the cells were provided with a differentiation medium consisting of DMEM-F12 without phenol red, 2% CS-FBS, 1 μM medroxyprogesterone acetate (MPA), and 0.5 mM 8-bromoadenosine-3',5'-cyclic monophosphate (cAMP) for 6 days. For comparison, undecidualized control wells were cultivated in the same medium without decidualization additives. The culture medium was semi-changed on day 3. After 6 days of differentiation, photographs were taken from each well, and the circularity index (CI) of undecidualized (uD) and decidualized (D) EnSCs was analyzed by image J (v1.52) and R (v4.3.3) software. Supernatants were collected and stored at -20 °C for prolactin measurement. According to the manufacturer’s instructions, the prolactin concentration was measured using an ELISA kit (Diazist, Iran). The expression of *PRL* and *IGFBP1* genes was assessed by quantitative real-time PCR (qRT-PCR) in reference to the *RPL19* housekeeping gene (the sequence of forward and reverse primers is supplied in supplementary Table S2) after RNA extraction (RNJia Kit, Rojetechnologies, Iran) and cDNA synthesis (RT-ROSET, recombinant M-MLV kit, Rojetechnologies).

**Assessment of HLA-G and Ki67 Expression by EnSCs**

EnSCs sourced from 4 to 6 patients were separately cultivated in 6-well culture plates in the presence or absence of recombinant human 25 ng/ml IFN-γ (Pepro Tech, USA) for 48h. Following this, cells underwent IVD for 6 days. Cells were then detached using a citrate buffer and labeled with purified mouse anti-human HLA-G antibody (BioLegend, cat no: 335902) and subsequently with FITC-conjugated sheep anti-mouse immunoglobulin (Sina Biotech). Signals were read by a flow cytometer (BD FACSLyric^TM^) after excluding propidium iodide (PI) positive dead cells. JEG3 cell line was used as the positive control. To evaluate the expression of Ki67, cultured EnSCs were either decidualized or remained undecidualized in a 6-day culture period. After that, the cultured cells were fixed using ice-cold acetone and labeled with mouse anti-human Ki67 antibody (Sina Biotech) and FITC-conjugated sheep anti-mouse immunoglobulin. DAPI was used for nuclear staining. The percentage of positive cells was determined in 10 HPF of an epifluorescent microscope (Olympus, BX51, Japan) and averaged.

**Metabolomics analysis of decidualized EnSCs**

In each well of 12-well culture plates, 1.5 × 10^5^ EnSCs were cultured in a final volume of 1 ml DMEM-F12 containing 10% FBS and subjected to IVD as above. Undifferentiated control wells were cultivated in the same medium without adding differentiation additives. The cell culture was sustained for 6 days, with medium semi-changes performed every 2 days. Supernatants were collected on days 2, 4, and 6 and aliquoted for various assessments, including the measurement of secreted PRL using an ELISA kit (Diazist, Iran), metabolomics analysis by targeted LC-MS/MS analysis using QSight 210 MD (Perkin Elmer, USA), quantification of glucose (Glu) and lactate (LAC) concentrations by glucose and lactate colorimetric assay kits (GLUC3 and LAC2, both from Roche diagnostics, USA), and the evaluation of IL-6 and IL-8 using ELISA kits (BioLegend). All of these assessments were carried out in accordance with the manufacturer’s instructions.

**Isolation and characterization of EnSCs-EV**

The isolation of extracellular vesicles derived from human endometrial stromal cells (hEnSCs-EV) was carried out using the EXOCIB C (Cibbiotech, Iran) or ByOMICs kits (Zist protein pajooh Tehran, Iran), according to the manufacturer's instructions. Cell culture supernatants were first concentrated using 30 KD filters (Merck Millipore, Germany) to increase EV yield, and the resulting concentrates were used for EV isolation. Both kits yielded the same results, so all EV isolation procedures were thereafter performed using the EXOCIB C kit. The isolated EVs were stored at -70°C. Culturing and decidualization of EnSCs were done as described above, except that charcoal-stripped exosome-free FBS (Gibco) was used to avoid contamination with FBS-derived exosomes. EnSCs were subjected to IVD for either 2, 4, or 6 days, and at each time point, the culture medium was collected for EV isolation. In 4 and 6 days IVD procedure, medium semi-change was done on days 2 and 3 of decidualization, respectively. A field emission scanning electron microscopy (FE-SEM) analysis was performed to visualize the ultrastructure of the isolated EVs. The size distribution and zeta potential of the freshly isolated EV diluted in PBS were assessed using a dynamic light scattering (DLS) (Nano ZS, Malvern Instruments, UK) at an ambient temperature of 23–28°C. These measurements were performed at least three times in three independent experiments. The protein concentration of isolated extracellular vesicles was determined using a BCA protein assay following the manufacturer’s instructions (Thermo Scientific, USA). The expression of TSG-101, CD81, and CD63 in isolated EV was assessed by Western blotting. In summary, 20 μg EV protein was mixed with 2x Laemmli sample buffer containing 10% 2-mercaptoethanol. The mixture was then boiled for 5 minutes, and SDS-PAGE electrophoresis was performed with 5% stacking and 12.5% resolving gels. The proteins were subsequently transferred onto a polyvinylidene fluoride (PVDF) membrane (Roche, USA). Afterward, the membrane was blocked for one hour at room temperature with 3% BSA in TBST (Tris-buffered saline with a pH of 7.5 and 0.05% Tween-20). Following a series of washes with TBST, the membrane was incubated overnight at 4°C with the optimal diluting of primary antibodies (CD63, CD81, and TSG101) (Santa Cruze, cat no: SC-5275, SC-166029, and SC-7964, respectively), diluted in 3% BSA in TBST. After 3-5 washes for 5 minutes with TBST, the membrane underwent incubation with a secondary antibody (Mouse anti-rabbit IgG-HRP) (Santa Cruze, cat no: SC-2357), diluted 1:1000 in 5% skim milk in TBST, for 1 hour at room temperature. Following another round of 3-5 washes for 5 minutes with TBST, the signals were developed using ECL (GE Healthcare, UK), and visualized on Kodak films.

**Assessment of EnSCs-EVs internalization**

EnSCs-EVs were labeled with PKH-26 red fluorescent cell linker kit (Sigma) according to the manufacturer’s instructions. Subsequently, the labeled EnSCs-EVs were subjected to a wash in DMEM-F12 by an Amicon filter (10 KD) (Merck Millipore). To assess the internalization of EnSCs-EVs by EnSCs, 5×10^4^ cells were plated on 6-well slides and incubated overnight until they reached a cell confluency of 60–70%. Subsequently, labeled EnSCs-EVs were introduced into each well at 25 μg/ml concentrations for 1, 3, or 4 hours. Cells were then fixed by ice-cold acetone and labeled with mouse anti-human vimentin antibody followed by FITC-conjugated sheep anti-mouse immunoglobulin and DAPI nuclear staining. Images were captured by an epifluorescent Olympus BX51 microscope equipped with a DP71 CCD camera. For NK cell internalization of EnSCs-EVs, purified NK cells were seeded in 24-well plates at a density of 1×10^6^ cells per well. Internalization of PKH-26-labeled EnSCs-EVs was then assessed by flow cytometry at 4 and 24-hour time points following the addition of the EVs.

**Isolation of human foreskin (FSK) stromal cells and FSK-EVs**

Foreskin tissues collected from children aged 2–6 months undergoing circumcision were transported to the lab in a cold DMEM-F12 culture medium containing penicillin (100 IU/ml) and streptomycin (0.1 mg/ml). Isolation of foreskin fibroblast was done according to the protocol published elsewhere with minor modifications ^1^. In brief, the tissues were treated with 5 ml 70% (v/v) ethanol for 5 minutes, then washed three times with PBS in a sterile Petri dish. Washed foreskin tissues were placed in 3 ml 4 mg/ml Dispase II (Sigma) and incubated at 4°C overnight. The epidermis was then carefully and thoroughly removed from the dermis, and dermal tissues were dissected into small pieces of approximately 1 mm³ using sterile scissors and forceps. Chopped tissues were incubated in 3 ml of 10 mg/ml Collagenase IV (Sigma) in a water bath at 37°C for 2 hours, with gentle shaking every 20 minutes. Collagenase activity was terminated by adding 3 ml of media supplemented with 10% FBS, and the mixture was filtered using a 40μm sterile cell strainer to separate the cells from tissue fragments. The filtered sample was centrifuged for 5 minutes at 870g at room temperature. Following centrifugation, the cell pellet was resuspended in 1 ml α-MEM medium supplemented with 10% FBS, Glutamax, and b-FGF (10 ng/ml) and cultured. When cells reached approximately 80% confluency, the medium was changed to a resting medium (DMEM-F12 without phenol red, 2% CS-FBS) and incubated for 4 days, after which the supernatant was collected for EV isolation.

**Induction of decidualization with EnSCs-EVs**

To evaluate the effects of EVs derived from EnSCs on EnSCs' decidualization, sources of well-decidualized EnSCs were selected based on prolactin secretion levels equal to or above the median during IVD (n=12). Cells were either *in vitro* decidualized (D-EnSCs) or remained undecidualized (uD-EnSCs) and EVs were collected from conditioned media on days 2, 4, and 6 of decidualization (termed as DEV2, DEV4, and DEV6 for D-EnSCs and uDEV2, uDEV4, and uDEV6 for uD-EnSCs). EVs collected from all 12 donors for each day were pooled and used as a single fraction for subsequent experiments. In the next step, well-decidualized EnSCs sources were seeded into 96-well plates at a density of 3×10^4^ cells per well (n=4). After reaching 70% confluency and the hormonal rest step, the EnSCs were co-cultured with 5-30 µg/ml EVs instead of receiving decidualization stimuli. To identify the optimal conditions for decidualization, we assessed the effects of EVs alone or in combination with additional decidualization stimuli, including E2 (10 nM), MPA (1 µM), and cAMP (0.5 or 0.05 mM). The composition of different combinations of decidualization stimuli were as follows: EVs alone, EVs + MPA + cAMP (0.5 mM), EVs + MPA, EVs + MPA + E2, EVs + MPA + E2 + cAMP (0.05 mM). To determine the kinetics of the effect of EVs, conditioned media from EV-treated EnSCs were collected on days 3, 6, and 9 of the culture period. Prolactin secretion levels were measured to assess the impact of EVs over time. For 6-day cultures, medium semi-change was done on day 3; for 9-day cultures, additional medium semi-change was performed on days 3 and 6 of decidualization. Semi-change media contained EVs with the same initial concentrations.

**NK cell purification and culture**

NK cells were purified from buffy coat samples from the Iran Blood Transfusion Organization (IBTO). In brief, mononuclear cells were isolated using Ficoll-Hypaque density gradient centrifugation (GE HealthCare, USA). NK cells were subsequently negatively selected using the NK cell isolation kit (Miltenyi Biotec, Germany) following the manufacturer's instructions. Flow cytometry was employed to confirm the purity of CD56^+^ NK cells. The optimal culture period and IL-15 concentration (BioLegend) for NK cell recovery, viability, and proliferation were determined through cell counting and trypan blue staining (Suppl. Fig. S2). Freshly isolated NK cells were cultured in 96-well plates containing RPMI-1640 (Gibco) supplemented with penicillin G at 100 IU/ml and streptomycin at 0.1 mg/ml and 10% FBS. This was carried out in the presence of varying concentrations (5-10-20 ng/ml) of recombinant human IL-15 for 3–7 days. Based on the results, concentration of 10 ng/ml of IL-15 for 5 days was selected as the optimal NK cell culture condition, and all subsequent experiments were conducted accordingly.

**NK cell Proliferation assay**

The effect of EVs derived from IFN-γ pre-treated EnSCs (uDEV4 and DEV4) on the proliferation of NK cells was investigated using four samples of NK cells obtained from different donors. Purified NK cells were cultured in a U-shaped bottom 96-well microplate (2×10^4^ cell/well), either with or without uDEV or DEV at concentrations of 5 and 30 μg/ml in RPMI-1640 supplemented with IL-15 (10ng/ml) for 5 days. Prior to the co-culture with uDEV and DEV, the NK cells were labeled with 5 μM CFSE (eBioscience, USA). The cultures were maintained for 5 days with a semi-change of the culture medium done on day 3, after which the NK cells were harvested, stained with Zombie Aqua^TM^ Fixable Viability Kit (BioLegend) and PE anti-human CD56 (Beckman Coulter, USA, cat no: A07788), and their proliferation was assessed using flow cytometry.

**NK cell cytotoxicity assay**

The cytotoxicity of NK cells was assessed in two distinct experiments, targeting either K562 cells or EnSCs, using the Calcein-AM (C-AM) (BD Bioscience) fluorimetric assay. For K562 cell cytotoxicity evaluation, freshly isolated NK cells were cultured either alone, treated with decidualization mediators (0.5 mM cAMP and 1µM MPA), or treated with EVs derived from IFN-γ pre-treated EnSCs (DEV4 or uDEV4) at a concentration of 30 μg/ml in complete RPMI-1640 medium supplemented with 10 ng/ml IL-15 and exosome-free FBS for 4 days. The culture medium was semi-changed on day 2. After a 4-day incubation period, NK cells were collected and co-cultured in duplicate with 5 μM C-AM-labeled K562 cells (10^5^ cells) as target cells at effector: target (E:T) ratio of 1:1 in 200 μL final volume in 96-well U bottom plates for 4 h. The spontaneous release of CAM was determined by incubating CAM-labeled K562 cells in medium alone, while target cells lysed with 2% Triton X-100 for 10 min served as controls for maximum release. After a 4-hour incubation at 37°C in 5% CO2, supernatants were collected, transferred to a 96-well black flat bottom polystyrene microplate (Corning, USA), and fluorescence intensity was measured using FLx800 microplate fluorescence reader (Bio-Tek, USA) at excitation/emission wavelengths of 488 nm/520 nm. Cytotoxicity, represented as the specific release percentage of C-AM, was calculated using the provided formula:

Percent Specific Release = (Experimental release - Spontaneous release) / (Maximum release - Spontaneous release) × 100.

Four different EnSC sources were utilized as targets to evaluate the cytotoxic effect of NK cells on EnSCs. EnSCs were seeded at 10^5^ cells/well in a 96-well flat clear bottom, black polystyrene TC-treated microplate (Corning). Co-culture of EnSCs with NK cells, either non-treated or pre-treated with 30 μg/ml EVs derived from IFN-γ pre-treated EnSCs (DEV4, uDEV4) for 4 days was conducted for 72 hours. After removal of NK cells and supernatant, adherent EnSCs were loaded with 1 μM C-AM for 30 minutes, and fluorescence intensity was measured. Negative control wells contained target cells cultured in medium alone, while positive control wells contained Triton X-100-lysed C-AM-labeled EnSCs. Corrected means were computed by subtracting the mean fluorescent signal of positive control wells from either co-cultured or negative control wells. The percentage of cytotoxicity was then calculated using the provided formula:

Percent Specific Release = 100 - (Co-culture cm / Control cm) × 100

**Analysis of NK degranulation and cytokine release**

To assess the regulatory effects of EnSCs-EVs (DEV, uDEV) on production of cytokines and cytotoxic proteins, NK cells were collected after 4 days treatment with EVs derived from IFN-γ pre-treated EnSCs (DEV4, uDEV4), and co-cultured with K562 cells (3×10^4^ cells) at an E:T ratio of 4:1 in U-bottom 96-well culture plates (SPL, Korea). Untreated NK cells served as negative control. In a parallel condition, NK cells were also treated with decidualization mediators (0.5 mM cAMP and 1µM MPA). After 4 hours, cells were harvested and stained with FITC-labeled anti-CD16 (Agilent Technologies, USA, cat no: F7011) and PE-labeled anti-CD56 (Beckman Coulter). Additionally, NK cells stained with APC-labeled anti-CD56 (BioLegend, cat no: 318310), were fixed and permeabilized using fixation and permeabilization buffer (BioLegend), followed by staining with FITC-labeled antibody against IFN-γ (BioLegend, cat no: 502506) or PE-labeled antibodies against perforin (BD Bioscience, cat no: 556437), granzymes A (BioLegend, cat no: 507206), and B (BD Bioscience, cat no: 561142). For CD107a expression, PE-labeled anti-CD107a (BD Bioscience, cat no: 555801) was added to NK-K562 co-cultures for 4 hours in the presence of Monensin (BioLegend). Afterward, cells were harvested and stained with APC-labeled anti-CD56 for flow cytometry analysis.

**Statistical analysis**

All statistical analyses and group comparisons were conducted using GraphPad Prism (v8.0) and R (v4.3.3). The data were compared using appropriate tests, including Wilcoxon, Mann-Whitney, and T-test. Correlation was evaluated using Spearman’s rank correlation and simple linear regression analysis. All clustering techniques and differential metabolite analyses were performed using MetaboAnalyst (v6.0)

Descriptive data were presented as the median or mean ± standard deviation (SD). Statistical analysis of the circularity index was performed using ImageJ (v1.52). P-values less than 0.05 were considered statistically significant. P-values less than 0.05, 0.01, 0.001, and 0.0001 are represented as *, **, ***, and ****, respectively.

**Supplementary References**

1. Jagadeeshaprasad MG, Govindappa PK, Nelson AM, Elfar JC. Isolation, culture, and characterization of primary Schwann cells, keratinocytes, and fibroblasts from human foreskin. *JoVE (Journal of Visualized Experiments)*. 2022;(181):e63776.

**Supplementary Figure Legends**

**Figure S1. Immunophenotypic characterization and optimizing culture condition of endometrial stromal cells (EnSCs).** EnSCs were isolated from endometrial biopsies and characterized. **A)** EnSCs expressed mesenchymal markers (CD10, CD73, and CD90) but failed to express hematopoietic markers (CD34 and CD45). Data from 3–4 donors was presented as mean percentage ± standard deviation (SD). **B)** EnSCs were *in vitro* decidualized using 8-Br-cAMP and MPA over 6 days (D) or remained undifferentiated (uD). Immunofluorescence analysis confirmed vimentin expression in both cell types but no detectable expression of cytokeratin was observed. Amniotic epithelial cells (AECs), as a positive control (PC), expressed high levels of cytokeratin. Nuclear staining was performed with DAPI. NC: Negative control. Scale bars: 100μm and 200μm. **C)** EnSCs cultured in α-MEM showed higher proliferation at different seeding densities compared to DMEM-F12. Supplementation of α-MEM with L-alanyl-L-glutamine (GlutaMAX-I), or GlutaMAX-I plus basic fibroblast growth factor (b-FGF), further increased the proliferation of EnSCs, especially at lower seeding densities.

**Figure S2. Characterization of decidualized EnSCs.** EnSCs were *in vitro* decidualized using 0.5mM 8-Br-cAMP and 1μM MPA for 6 days (D) or remained undifferentiated (uD). **A)** Expression of decidualization transcripts, *PRL* and *IGFBP1* increased several folds compared to undifferentiated cells. **B)** Decidualized EnSCs also secreted high levels of prolactin in their culture supernatants. **C)** Expression of *PRL* transcript correlated with the secretion of prolactin by decidualized EnSCs. **D)** EnSCs were in *vitro* decidualized using standard (cAMP 0.5mM, MPA 1μM) or 2X (cAMP 1mM, MPA 2μM) concentration of cAMP and MPA. Results showed the negative impact of higher concentration of decidualization stimuli on secretion of prolactin by decidualized EnSCs at both day 3 and 6 (n = 4). **E)** Microscopic images of uD and D cells were captured after 6 days of decidualization. **F)** D cells exhibited higher circulatory index and lower proliferative capacity **(G)** compared to uD cells, as assessed by Ki67 immunofluorescent staining. **H)** The levels of glucose (Glu) and lactate (LAC) in the cell culture supernatants were measured on decidualization days 2, 4 and 6 using colorimetric assay kits (n = 7). Based on the results, there was an inverse correlation between lactate and prolactin (PRL) levels on decidualization day 6. However, no significant correlation was found between glucose level and PRL secretion on decidualization days 2, 4, and 6. **I)** The levels of IL-8 and IL-6 in cell culture supernatants were determined on decidualization days 2, 4 and 6 using enzyme-linked immunosorbent assay (ELISA) (n = 7). Correlation analysis determined a significant inverse relationship between decidualization status (PRL levels) and inflammatory cytokine concentrations on days 2, 4, and 6 of decidualization. Scale bars: 100μm. P-values less than 0.05, 0.01, 0.001, and 0.0001 are represented as *, **, ***, and ****, respectively.

**Figure S3. Yield and size distribution of extracellular vesicles (EVs) derived from EnSCs.** EVs were isolated from EnSCs and characterized. EVs were isolated using two extraction kits (EXOCIB C and ByOMICs). The EV yield **(A)** and size distribution **(B)** were assessed using protein quantification (BCA assay) and dynamic light scattering (DLS) methods, respectively across three independent experiments, both kits isolated EnSCs-EVs with the same yield and size of about 80 nm. **C)** The variation in protein content of EnSCs-derived EVs isolated using the EXOCIB C kit was evaluated across four independent experiments. The BCA assay revealed that the protein content of EnSCs-EVs accounted for approximately 3.81% of the total protein present in the initial cell culture supernatant.

**Figure S4. Effect of IFN-γ pre-treatment on HLA-G expression and decidualization of EnSCs.** EnSCs were *in vitro* decidualized using 0.5mM 8-Br-cAMP and 1μM MPA for 6 days (D) or remained undifferentiated (uD). **A)** D or uD cells, were either pre-treated with 25 ng/ml IFN-γ for 48 hours before decidualization or remained untreated. After 6 days, HLA-G expression was analyzed by flow cytometry (n = 4-6). Pre-treatment of D cells with IFN-γ caused a significant increase in HLA-G expression. JEG3 cells served as a positive control. **B)** D or uD cells pre-treated with 10 ng/ml IFN-γ were evaluated for PRL secretion on days 3 and 6 using enzyme-linked immunosorbent assay (ELISA). At both days, IFN-γ pre-treatment significantly inhibited prolactin secretion by decidualized EnSCs (n = 4). **C)** To assess the impact of inflammatory signaling on EnSCs differentiation, EnSCs were pre-treated with 10 ng/ml IFN-γ or remained untreated before *in vitro* decidualization, and EVs were isolated on day 4 of decidualization. The effect of these EVs on the decidualization of EnSCs stimulated with EPC was then assessed. The results showed that IFN-γ pre-treatment, caused a lower pro-decidualization potency of DEVs. EPC: 10nM E2 + 1μM MPA + 0.05mM cAMP. P-values less than 0.05, 0.01, 0.001, and 0.0001 are represented as *, **, ***, and ****, respectively.

**Supplementary Table Legends**

**Table S1. The size of extracted EVs using EXOCIB C and ByOMICS kits in three separate experiments.**

**Table S2. Primer sets used in this study.**
