# Supplementary material for: Decidualised endometrial stromal cell‐derived extracellular vesicles induce bystander decidualisation and cAMP‐mediated attenuation of natural killer cell cytotoxicity
Source: Clin Transl Med. 2025 Oct 11;15(10):e70500. doi: 10.1002/ctm2.70500 (PMC12514555; doi:10.1002/ctm2.70500)
Supplement: Supplementary file 6 — Supporting Information [file CTM2-15-e70500-s004.docx]

**Table S1. The size of extracted EVs using EXOCIB C and ByOMICS kits in three separate experiments.**

| **Size(nm) ±SD**  **(ByOMICS)** | **Size(nm) ±SD**  **(EXOCIB C)** | **Experiment** |
| --- | --- | --- |
| 80 ± 3.756 | 77.71 ± 3.275 | 1 |
| 80.36 ± 4.098 | 77.83 ± 3.115 | 2 |
| 81.27 ± 4.949 | 76.99 ± 4.044 | 3 |
