# Supplementary material for: Decidualised endometrial stromal cell‐derived extracellular vesicles induce bystander decidualisation and cAMP‐mediated attenuation of natural killer cell cytotoxicity
Source: Clin Transl Med. 2025 Oct 11;15(10):e70500. doi: 10.1002/ctm2.70500 (PMC12514555; doi:10.1002/ctm2.70500)
Supplement: Supplementary file 7 — Supporting Information [file CTM2-15-e70500-s003.docx]

**Table S2. Primer sets used in this study.**

| **Gene** | **Primer sequence** | **Product length (bp)** |
| --- | --- | --- |
| *RPL19 (L19, eL19)* ^†^ | 5^’^-GCGGAAGGGTACAGCCAAT-3^’^  5^’^-GCAGCCGGCGCAAA-3’ | 77 |
| *PRL*^‡^ | 5^’^-AAGCTGTAGAGATTGAGG AGCAAAC-3’  5^’^-TCAGGATGAACCTGGCTGACTA-3’ | 76 |
| *IGFBP-1*^§^ | 5^’^-CGAAGGCTCTCCATGTCACCA-3’  5^’^-TGTCTCCTGTGCCTTGGCTAAAC-3’ | 98 |

† Ribosomal Protein L19, ‡ Prolactin, § Insulin-like Growth Factor Binding Protein 1
